# Age-Specific Ex Vivo Modulation of Gut–Brain Axis-Associated Metabolites by Galacto-Oligosaccharides and Nutrient Blends in Early Childhood

**DOI:** 10.3390/metabo16040255

**Published:** 2026-04-10

**Authors:** Laurent Ferrier, Shaillay Kumar Dogra, Lam Dai Vu, Alexandros K. Kanellopoulos, Jonas Poppe, Laurence Biehl, Aurélien Baudot, Pieter Van den Abbeele

**Affiliations:** 1 Nestlé Institute of Health Sciences, Nestlé Research, Route du Jorat 57, 1000 Lausanne, Switzerland; 2 Cryptobiotix SA, Technologiepark-Zwijnaarde 82, 9052 Ghent, Belgium; 3 Nestlé Product Technology Center, Nestlé Strasse 3, 3510 Konolfingen, Switzerland

**Keywords:** GOS, gut–brain axis, microbiome, neuroactives, vitamin, amino acid, mineral, synergistic effects

## Abstract

**Background**: Gut microbiome-derived metabolites, particularly short-chain fatty acids (SCFA) and tryptophan derivatives, are central mediators of the gut–brain axis. This ex vivo study assessed how nutritional interventions impact such metabolites during early life, a critical period for neurodevelopment. **Methods**: The effects of galacto-oligosaccharides (GOS), nutrient blends (vitamins, minerals and amino acids) and their combinations were evaluated in the gut microbiomes of infants (2–4 months, *n* = 6) and young children (2–3 years old, *n* = 6) using the ex vivo SIFR^®^ technology. **Results**: Baseline microbiome composition was age-dependent, with infants displaying lower α-diversity and greater interpersonal variability. After ex vivo incubation, nutrient blends increased the propionate/butyrate ratio and branched-chain fatty acids in young children and elevated several B-vitamins and amino acid-derived metabolites, including indole-3-carboxaldehyde, imidazoleacetic acid and pipecolinic acid. Combining nutrient blends with GOS exhibited potential synergistic effects on propionate (infants) and 2-hydroxyisocaproic acid (HICA, both age groups). GOS strongly stimulated *Bifidobacteriaceae* and increased metabolites linked to bifidobacterial metabolism like acetate, HICA, N-acetylated amino acids, aromatic lactic acids and acetylagmatine; in young children, butyrate and γ-aminobutyric acid (GABA) also increased. **Conclusions**: Combinations of GOS with nutrient blends impacted microbiome-derived metabolites associated with the gut–brain axis, with potential synergistic increases of metabolites with emerging roles in neurodevelopment, including GABA, acetylagmatine and HICA. Despite shared bifidogenic effects, differences between age groups indicate that microbiome maturity may influence responses to nutritional intervention. Future clinical studies are needed to determine whether these metabolite changes translate into neurodevelopmental benefits in vivo.

## 1. Introduction

The gut–brain axis describes bidirectional communication between the gastrointestinal tract and the central nervous system through neural, endocrine, immune and humoral pathways [1]. Mechanistically, brain function is impacted by microbial metabolites like short-chain fatty acids (SCFA, e.g., acetate, propionate and butyrate) [2] and tryptophan-derived metabolites (e.g., indoles) [3], along with microorganism-associated molecular patterns (MAMP, e.g., nucleotides, lipids, carbohydrates and peptides) [4]. Conversely, the brain can influence gut physiology and function by regulating immune responses and controlling the release of endocrine hormones and neurotransmitters. These signaling molecules travel through systemic circulation, affecting gut motility, immune activity and microbial composition [5].

The first few years are crucial for neurodevelopment [6] and are a period during which the gut microbiome also undergoes drastic alterations, along with others that are driven by the mode of delivery, type of feeding (breast or formula) and use of antibiotics [7]. While the infant microbiome has a low α-diversity and mainly consists of *Bifidobacteriaceae*, *Bacteroidaceae* and *Enterobacteriaceae*, the microbiota of young children is highly diverse and dominated by adult-like taxa such as *Bacteroidaceae*, *Lachnospiraceae* and *Ruminococcaceae* [8]. Deviations in gut microbiome composition in early life have been associated with autism spectrum disorder [9], emphasizing a window of opportunity for nutritional interventions in early life to prevent neurological and mental diseases by impacting the gut microbiome.

Probiotics are live microorganisms that can confer health benefits when administered in adequate amounts [10]. Early-life probiotic supplementation has been suggested to influence neurodevelopmental outcomes, as illustrated by studies linking *Lactobacillus rhamnosus* GG administration during infancy to a reduced incidence of certain neuropsychiatric conditions later in childhood [11]. Prebiotics, in contrast, are non-digestible substrates selectively utilized by host microorganisms [12] that exert benefits by modulating the indigenous gut microbiota. Galacto-oligosaccharides (GOS) are among the most widely used prebiotics in infant nutrition and are known to stimulate the growth of health-related *Bifidobacterium* species [13,14,15,16]. Such bifidogenic shifts promote the production of microbial metabolites, including SCFAs, acetylated amino acids and GABA, all of which are increasingly recognized for their roles in neurodevelopment.

In addition to probiotics and prebiotics, infant nutrition also provides vitamins, amino acids and minerals [17]. B-vitamins (such as B1, B2 and B6) [17] act as cofactors in neurotransmitter synthesis, while amino acids like tryptophan, tyrosine, leucine, lysine and histidine are precursors to neuroactive compounds such as serotonin, melatonin, dopamine, GABA, acetylagmatine and pipecolinic acid. Although these nutrients are essential for normal development, their potential interactions with the gut microbiome during early life remain incompletely understood.

This study aimed to assess how GOS, nutrient blends (of vitamins, minerals and amino acids) and combinations thereof impact the gut microbiome of infants (2–4 months old) and young children (2–3 years old). The selection of specific micronutrients and amino acids included in the nutrient blends was based on previous work by our group, which showed that the intake of a combination of vitamins B1, B2, B6, zinc, copper, iron, histidine, isoleucine, lysine and leucine was positively correlated with levels of myelination in social brain areas in infants [18]. This prior evidence provided a strong rationale for testing these ingredients, not only for their nutritional value but also for their potential to influence microbiome-derived metabolites associated with neurodevelopment. To obtain mechanistic insights, we used the ex vivo SIFR^®^ technology, a high-throughput, miniaturized, bioreactor-based model that accurately cultivates the gut microbiome in the laboratory. Importantly, this technology has been validated to provide clinically predictive insights in microbiological [19] and host endpoints [20]. 

## 2. Materials and Methods

### 2.1. Test Products

GOS and nutrient blends (BL for infants, BL+ for young children) were provided by Nestlé Research (Lausanne, Switzerland). The composition and test doses of the nutrient blends are provided in Table 1. GOS was tested at 4 g/L. The selection of micronutrients and amino acids included in the nutrient blends was based on previous work demonstrating associations between their intake and early neurodevelopmental outcomes [18]. The dose levels were defined based on the mean daily intake levels corresponding to the respective age groups identified in that study.

### 2.2. Fecal Donor Selection Criteria

Fecal samples were collected according to a procedure approved by the Ethics Committee of the University Hospital Ghent (reference number BC-09977; approval date 13 April 2021). Parents of infants and young children signed an informed consent in which they donated the fecal sample of their child for this current study.

Inclusion criteria for 2–4 month-old infants were exclusive formula-feeding (without human milk oligosaccharides), while 2–3 year-old young children had to comply to an omnivorous diet. Exclusion criteria were antibiotic use in the past 3 months and gastrointestinal disorders (cancer, ulcers, IBD). This resulted in the enrolment of 12 specific subjects (*n* = 6 per age group) with an average age of 3.0 (±0.6) and 29.5 (±3.3) months for infants and young children, respectively.

While key dietary inclusion criteria were applied, detailed demographic and clinical information (e.g., ethnicity, delivery mode, recent health status and specific dietary patterns) was not systematically recorded. These factors may contribute to interindividual variability in microbiome composition.

### 2.3. Ex Vivo Intestinal Fermentation Assay (SIFR^®^) Design

**Figure 1:**
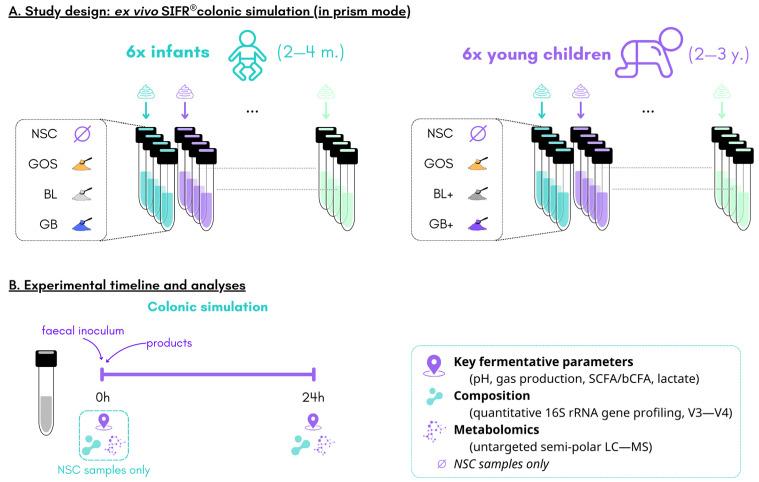
**Experimental configuration of the current study.** The impact of GOS, nutrient blends (BL (infants), BL+ (young children)) and combinations thereof (GB (infants), GB+ (young children)) was assessed in the gut microbiome of infants and young children using the ex vivo SIFR^®^ technology, compared to a parallel control (NSC). (**A**) Study design along with (**B**) timeline and analysis.

Four study arms were evaluated for each age group, i.e., GOS, nutrient blends (BL (infants), BL+ (young children)), the combination thereof (GB (infants), GB+ (young children)) and an unsupplemented parallel control with a minimal growth medium (NSC, no substrate control) (Figure 1A). This NSC served as a reference for evaluating treatment effects as it is identical to the treatments (same microbiome, same nutritional medium), except for the presence of GOS and/or blends. No adjustment was made to match the carbon load of the treatment conditions, as introducing alternative substrates in the control could confound the interpretation of treatment-specific effects.

Colonic fermentation was studied using a standardized ex vivo fermentation system with the SIFR^®^ technology as previously described [19]. Briefly, individual stool samples were processed in a bioreactor management device (Cryptobiotix, Ghent, Belgium). Each bioreactor contained 5 mL of a mixture of nutritional medium (M0019 (infants) or M0024 (young children), Cryptobiotix, Ghent, Belgium), fecal inoculum and the test product. After being rendered anaerobic, bioreactors were incubated under continuous agitation (140 rpm) at 37 °C. Upon gas pressure measurement, samples were collected at 0 h and 24 h for measurement of key fermentative parameters (pH, gas, SCFA, BCFA and lactate production), microbial composition and in-depth metabolite analysis (Figure 1B).

Control samples (NSC) were run in technical triplicate for each infant and child with analysis of key fermentative parameters confirming coefficients of variation below 3% illustrating the high technical reproducibility of the SIFR^®^ technology.

### 2.4. Key Fermentative Parameters

As previously described [21], after extraction in diethyl ether, SCFA (acetate, propionate, butyrate) and branched-chain fatty acids (BCFA; sum of isobutyrate, isovalerate and isocaproate) were determined via gas chromatography with flame ionization detection. Lactate was quantified using an enzymatic method (Enzytec^TM^, R-Biopharm, Darmstadt, Germany). Finally, pH was measured using an electrode (Hannah Instruments Edge HI2002, Temse, Belgium).

### 2.5. Taxonomic Microbiota Analysis by Quantitative 16S rRNA Gene Profiling

Quantitative insights were obtained by correcting proportions (%; 16S rRNA gene profiling) with total counts (cells/mL; flow cytometry), resulting in estimated cells/mL of different phyla, families and OTUs (operational taxonomic units), as recently described [20]. Briefly, DNA was extracted using the SPINeasy DNA Kit for Soil (MP Biomedicals, Eschwege, Germany), according to the manufacturer’s instructions. Subsequently, library preparation and sequencing were performed on an Illumina MiSeq platform with v3 chemistry. 16S rRNA gene V3–V4 hypervariable regions were amplified using primers 341F and 785Rmod. To determine total bacterial cell density, samples were diluted and subsequently stained with SYTO 16, after which cells were counted via a BD FACS Verse flow cytometer (BD, Erembodegem, Belgium).

### 2.6. Untargeted Metabolite Profiling

As previously described [22], liquid chromatography–mass spectrometry (LC–MS) analysis was carried out on a Thermo Scientific Vanquish LC coupled to Thermo Q Exactive HF MS (Thermo Fisher Scientific, Waltham, MA, USA) using an electrospray ionization source, both in negative and positive ionization mode. The data analysis focused on metabolites with a high degree of annotation confidence, based on a combination of retention time matching (using in-house authentic standards), accurate mass (with an accepted deviation of 3 ppm) and MS–MS spectral information where available. To focus on microbiome-derived metabolites, the main statistical analysis was restricted to metabolites that showed an increase in signal intensity from 0 h (NSC) to 24 h (in at least one treatment condition) in at least four out of six test subjects within a given age group. This approach ensured that only metabolites consistently produced during incubation were included in the analysis.

### 2.7. Data Analysis

All analyses were performed using R (version 4.2.2; www.r-project.org; accessed on 4 November 2024). R software was used to perform principal component analysis (PCA) and make violin plots and heat maps. For the key fermentative parameters and metabolomics analysis, significance of treatment effects was assessed via repeated-measure ANOVA analyses (based on paired testing among the six subjects per age group), with *p*-value correction according to Benjamini–Hochberg [23]. For the statistical analysis of microbial composition, three measures were taken. First, the analysis was performed on log_2_-transformed values. Second, a value of a given taxonomic group below the limit of detection (LOD) was considered equal to the LOD, as previously described [19]. Finally, paired *t*-tests were performed on the 50 and 100 most abundant OTUs, which covered on average 99.8% and 98.8% of the abundances of a given sample for infants and young children, respectively. An adjusted *p*-value threshold of 0.20 was applied to capture changes in microbial composition and metabolite production in this exploratory, hypothesis-generating study. This more lenient threshold was chosen given the modest sample size (*n* = 6) and the high dimensionality of the data, but this increased the risk of false-positive findings. To unravel microbial taxa responsible for the production of specific metabolites, Regularized Canonical Correlation Analysis (rCCA) was performed using the mixOmics package with the shrinkage method for estimation of penalization parameters (version 6.20.3) [24].

## 3. Results

### 3.1. Microbiome Composition Reflected Expected Age-Related Differences

**Figure 2:**
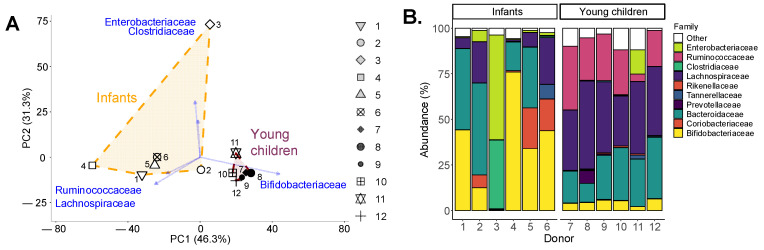
**Microbiome composition was highly age-dependent, with profound interpersonal differences among infants, contrasting with the adult-like microbiota of young children**. (**A**) PCA based on centered log_2_-transformed abundances of families (%), highlighting the five families that explained most variation in the dataset. Each of the infants and young children is indicated by a unique symbol. (**B**) Abundances (%) of most abundant families.

As expected, based on the previous literature, clear age-dependent differences in fecal microbiome composition were observed, with α-diversity (Chao1 diversity index) being considerably lower for infants (33.0 ± 11.2) compared to young children (128.1 ± 19.7). Further, interpersonal differences in fecal microbiome composition were noted amongst infants (Figure 2A), with *Bifidobacteriaceae* levels being as abundant as 75% of the community for infant 4 while being absent for infant 3, whose microbiome consisted of *Enterobacteriaceae* and *Clostridiaceae* (Figure 2B). In contrast, interpersonal differences among young children were smaller, with dominant community members belonging to the *Bacteroidaceae*, *Lachnospiraceae* and *Ruminococcaceae* families. The *Bifidobacteriaceae* family was consistently present for young children at levels from 2.2 to 6.2%.

### 3.2. GOS-Containing Treatments Stimulated SCFA, While Nutrient Blends Enhanced BCFA

**Figure 3:**
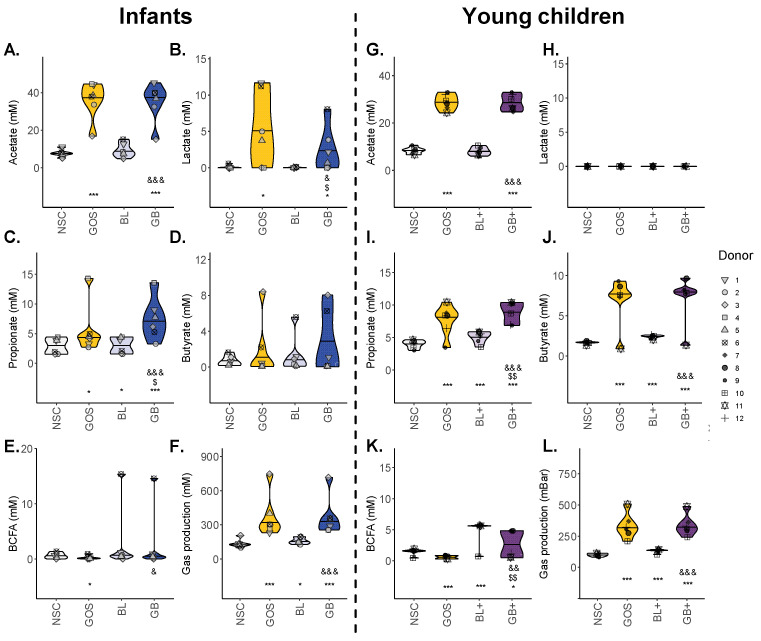
**GOS-containing treatments (GOS, GB, GB+) stimulated SCFA, while nutrient blends (BL, BL+) enhanced BCFA (mainly for young children).** SCFA/BCFA/lactate (mM) and gas production (mbar) for (**A**–**F**) infants and (**G**–**L**) young children upon treatment with nutrient blends (BL, BL+) and combinations thereof (GB, GB+) compared to an unsupplemented control (NSC). Data from each infant and young child are represented by unique symbols. Statistical differences with NSC are indicated with * (0.10 < p_adjusted_ < 0.20), ** (0.05 < p_adjusted_ < 0.10) or *** (p_adjusted_ < 0.05). ‘$’ indicates differences between GOS and GB/GB+, while ‘&’ indicates differences between BL/BL+ and GB/GB+.

GOS was well fermented by the microbiota of infants and young children, as followed from the stimulated acetate levels for both age groups (especially infants) (Figure 3A,G). While GOS also enhanced propionate for both age groups (Figure 3C,I), it increased lactate specifically for infants (Figure 3B) and butyrate specifically for young children (Figure 3J).

Additionally, the nutrient blend BL+ significantly stimulated propionate, butyrate and especially BCFA (predominantly isovalerate), while BL only mildly but still significantly increased propionate production for the infants (Figure 3).

Combinations of GOS and BL or BL+ (GB or GB+) resulted in the highest propionate and butyrate levels. While effects were mostly additive, the impact of GB on propionate levels for infants revealed a potential synergy between GOS and BL as the impact of GB exceeded the sum of the individual effects of GOS and BL (Figure 3C).

Finally, two remarkable interpersonal differences in butyrate production were noted for infants (Figure 3D): GOS or GB strongly increased butyrate for infant 3, while butyrate along with BCFA were especially notable with BL or GB for infant 6 (Figure 3E). The increase in bCFA was driven by both isovalerate and isobutyrate at comparable levels.

### 3.3. GOS and Nutrient Blend Altered Gut Microbiome Composition of Infants and Young Children in a Product-Specific Manner

**Figure 4:**
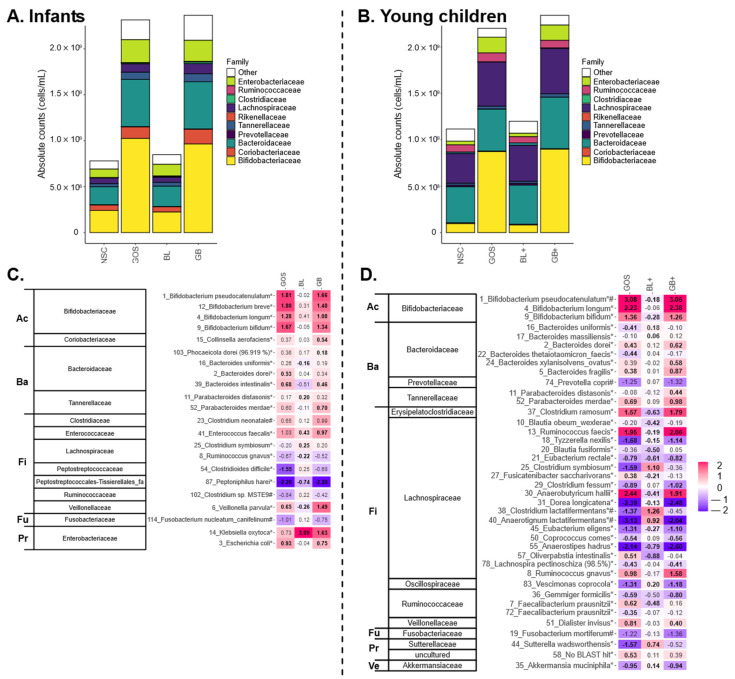
**GOS strongly impacted gut microbiome composition, strongly enhancing *Bifidobacteriaceae*, both in absence (GOS) and presence of nutrient blends (GB, GB+).** (**A**) Absolute levels (cells/mL) of the most abundant families for infants and (**B**) young children upon treatment with nutrient blends (BL, BL+) and combinations thereof (GB, GB+) compared to an unsupplemented control (NSC). Heatmap for (**C**) infants and (**D**) young children based on OTUs that were significantly (*p* < 0.20) affected by any of the treatments (highlighted with ‘*’) or that explained most variation in a PCA analysis (highlighted with ‘#’; PCA not shown), expressed as log_2_ (treatment/NSC), averaged over all test subjects within an age group. Values indicated in bold show significant increases (>0) or decreases (<0). Phyla to which the families belong are indicated on the left (Ac = Actinobacteriota, Ba = Bacteroidota, Fi = Firmicutes, Fu = Fusobacteriota, Pr = Proteobacteria, Ve = Verrucomicrobiota).

**Figure 5:**
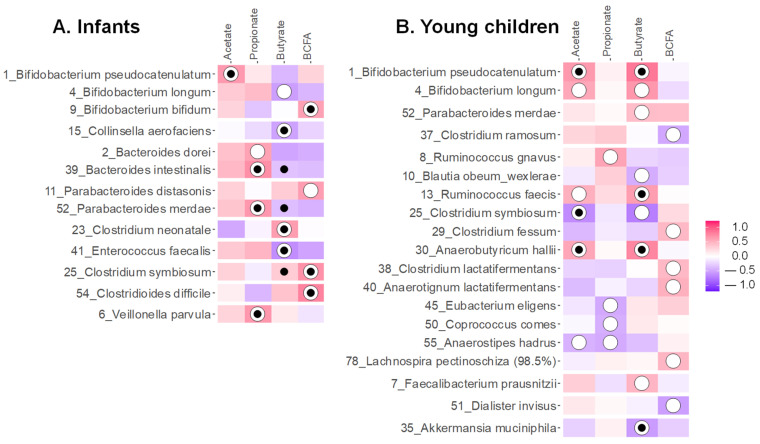
Regularized canonical correlation analysis (rCCA) to highlight correlations between SCFA or BCFA and microbial composition across all study arms for (**A**) infants (threshold = 0.46) and (**B**) young children (threshold = 0.41). The white circles indicate values larger than the threshold and black dots indicate statistical significance (*p* < 0.05) in the individual correlations between the SCFA and the OTUs based on Spearman’s rank correlation coefficient.

GOS, both with an absence and presence of nutrient blends (GOS, GB, GB+), impacted the microbiota of both age groups by boosting *Bifidobacteriaceae* (Figure 4A,B). This followed from the stimulation of OTUs related to a broad range of species, including *B. longum*, *B. bifidum* and especially *B. pseudocatenulatum* (Figure 4C,D), which correlated to elevated acetate levels upon GOS treatment (Figure 5A,B). Supplementary tables provide the absolute levels of families and OTUs (Table S1), the statistical output at OTU level (Table S2) and correlations for the three main SCFAs and bacterial OTUs shown in Figure 5 (Table S3).

For infants, GOS-containing products also promoted *Bacteroidaceae*, *Tannerellaceae* and *Veillonellaceae* (Figure 4A) due to increases of OTUs related to *Bacteroides dorei*, *Bacteroides intestinalis*, *Parabacteroides merdae* and *Veillonella parvula* (Figure 4C) that correlated with elevated propionate levels (Figure 5A). GOS or GB boosted butyrate for one infant (infant 3) related to potent increases of *Clostridium neonatale* for this specific infant. Other than stimulating various taxa, GOS-containing treatments lowered the abundance of, amongst others, an OTU related to pathogenic *Clostridioides difficile*.

For young children, GOS and its combination with the nutrient blend (GB+) stimulated OTU related to *Ruminococcus gnavus*, which was associated with elevated propionate production and *Bifidobacterium pseudocatenulatum*, *Ruminococcus faecis* and *Anaerobutyricum hallii*—all three correlating with increased butyrate levels (Figure 4D and Figure 5B). Further, *Faecalibacterium prausnitzii* (OTU7), which was promoted by GOS, was also positively correlated with butyrate in the rCCA analysis (Figure 4D and Figure 5B).

For both donor groups, GOS also increased *Enterobacteriaceae* members, an effect that was mainly driven by increases for test subjects where GOS did not exert bifidogenic effects, i.e., infant 3 and child 11, who had no or low *Bifidobacteriaceae* levels at baseline (Figure 2).

The nutrient blends (BL and BL+) exerted milder effects. The strongest effects were noted for young children for which BL+ increased the abundance of OTUs related to *Anaerotignum lactatifermentans* and *Clostridium lactatifermentans* (Figure 4D), which were related to elevated BCFA levels (Figure 5B). Additionally, in infants’ microbiota, BL significantly increased *Clostridium symbiosum* (OTU25) (Figure 4C), which positively correlated to butyrate based on Spearman’s rank correlation coefficient analysis (Figure 5A).

### 3.4. GOS and Nutrient Blends Altered the Gut Metabolome of Infants and Young Children

**Figure 6:**
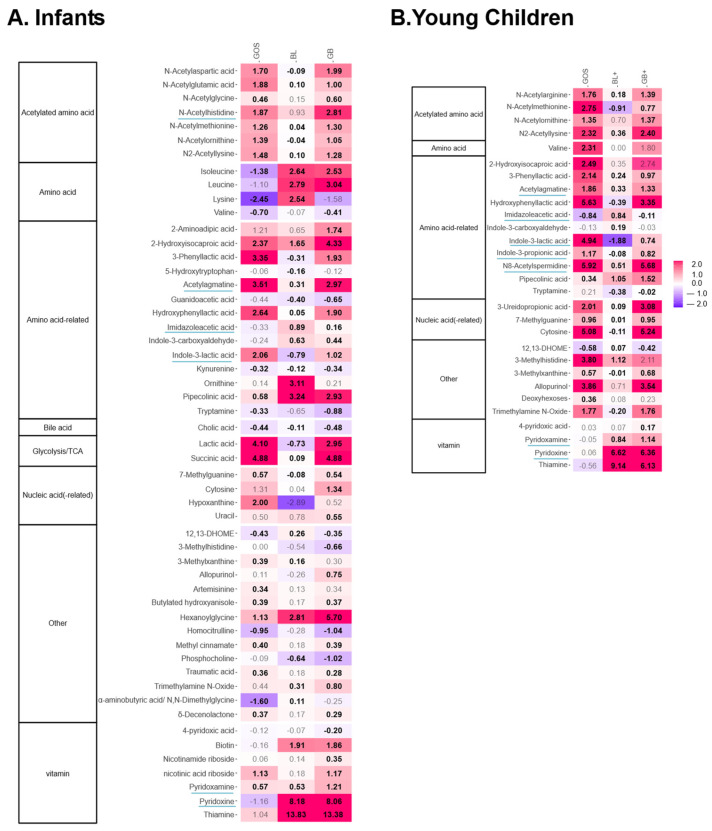
**GOS, nutrient blends (BL, BL+) and combinations thereof (GB, GB+) strongly impacted the gut metabolome.** Heatmap based on level 1 or 2a-annotated metabolites (detected via untargeted metabolite profiling) that were significantly affected by any of the treatments, expressed as log_2_ (treatment/NSC), averaged over all test subjects within an age group, i.e., (**A**) infants and (**B**) young children. Values indicated in bold show significant increases (>0) or decreases (<0) (*p* < 0.20). Neuroactive or neuroprotective metabolites discussed in the text are underlined.

**Figure 7:**
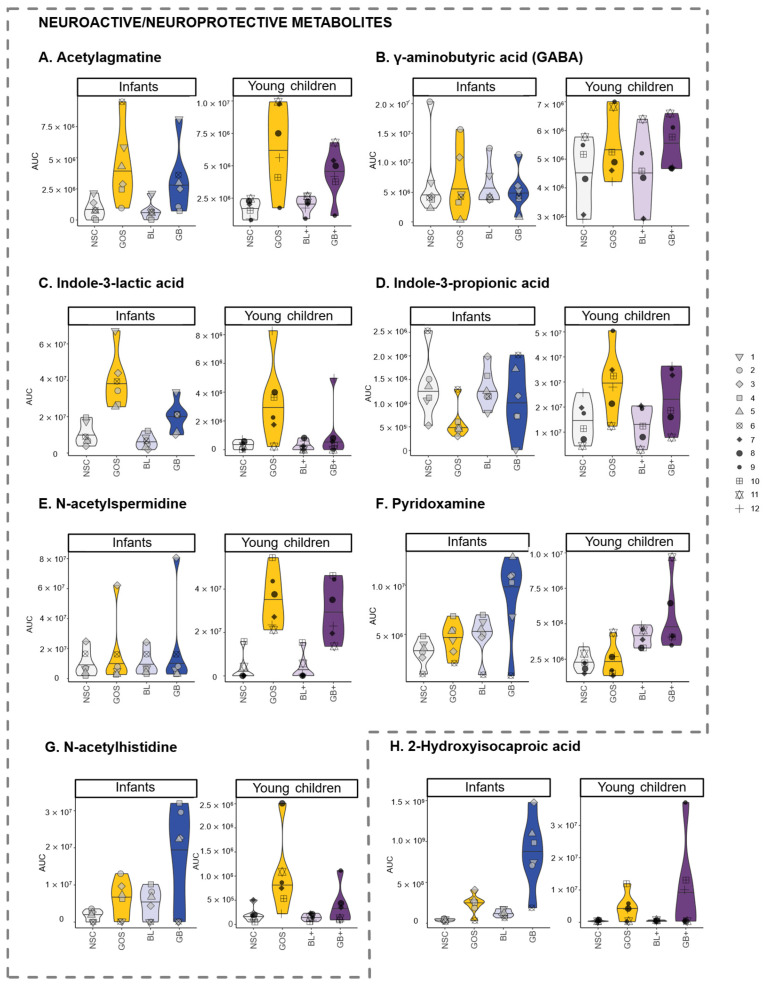
**GOS-stimulated neuroactive and neuroprotective compounds**. (**A**) Acetylagmatine, (**B**) GABA, (**C**) indole-3-lactic acid, (**D**) indole-3-propionic acid, (**E**) N-acetylspermidine, (**F**) pyridoxamine. Potential synergistic effects of combinations of GOS with nutrient blends (GB, GB+) were noted for (**F**) pyridoxamine, (**G**) N-acetylhistidine and (**H**) 2-hydroxyisocaproic acid. Absolute levels of these metabolites (AUC = area under the curve), as detected via untargeted metabolite profiling. Data from each infant and young child are represented by unique symbols.

An in-depth metabolomic analysis confirmed marked effects on metabolite production (Figure 6 and Table S4 (statistical output)). In both age groups, GOS exerted the strongest impact and promoted health-related metabolites derived from amino acid fermentation, including 2-hydroxyisocaproic acid (HICA), N-acetylated amino acids, aromatic lactic acids (3-phenyllactic acid, hydroxyphenyllactic acid, indole-3-lactic acid), N-acetylspermidine and indole-3-propionic acid (young children only), alongside two additional neuroactive compounds, i.e., acetylagmatine and GABA (young children only) (Figure 7). While GABA did not reach statistical significance after multiple testing correction, it showed a consistent increase across all six infants.

Further, for both age groups, the nutrient blends BL and BL+ increased metabolites such as indole-3-carboxaldehyde [25], imidazoleacetic acid [26,27] and pipecolinic acid [28,29] (Figure 6). In addition, levels of different B vitamins, i.e., biotin (infants only), pyridoxine (vit B6) and thiamine (vit B1), were also elevated (Figure 6). Strikingly, potential synergistic effects between nutrient blends and GOS were noted for HICA (GB or GB+), N-acetylhistidine (GB) and pyridoxamine (GB) (Figure 7F–H).

It should be noted that not all supplemented compounds included in the nutrient blends are shown in Figure 6. Only metabolites that exhibited an increase in signal intensity during incubation were retained, reflecting microbiome-derived production, whereas compounds that remained unchanged or were consumed were not included.

## 4. Discussion

### 4.1. Summary of Main Findings in Perspective to the Literature

First, infants and young children displayed clear age-related differences in fecal microbiome composition, in line with previous studies: while the infant microbiome was predominantly colonized with *Bacteroidaceae*, *Enterobacteriaceae* and particularly *Bifidobacteriaceae*, young children had more adult-like microbiota characterized by a high diversity of microbes mostly belonging to the *Bacteroidaceae*, *Lachnospiraceae* and *Ruminococcaceae* [7,8]. Interindividual variability was particularly pronounced in the infant cohort, which is consistent with the well-established heterogeneity of early-life microbiome development [30]. These strong interpersonal differences enabled obtaining representative insights into how GOS, blends and combinations thereof impact the microbiome of infants and young children.

Age-related and interpersonal differences impacted the outcome of the treatments. First, the stimulation of propionate, butyrate and BCFA production by the nutrient blends was only consistent (and significant) for young children, demonstrating that its more mature microbiota allows for better fermentation of the nutrient blends. Key constituents of the blends were lysine, which can indeed be converted to propionate [31] and butyrate [32], and branched chain amino acids (leucine, isoleucine), which are known to be metabolized to isovaleric acid [32] (main BCFA detected during the current study). A key species stimulated by nutrient blends for young children was *Anaerotignum lactatifermentans* (formerly *Clostridium lactatifermentans*), which has indeed been shown to ferment leucine or isoleucine to BCFA [33]. This highlights how age-related differences in microbiome composition affect treatment outcomes, stressing the importance of developing age-tailored nutritional interventions.

Another age-related finding was that propionate was strongly promoted by the combination of GOS with the nutrient blend for infants. As reviewed by Silva et al. (2020) [2], SCFA like propionate impact gut–brain communication via multiple pathways, e.g., (i) influencing immunity and gut barrier integrity upon binding to G protein-coupled receptors, (ii) interacting with receptors on enteroendocrine cells to induce the secretion of gut hormones thereby promoting signaling to the brain, or (iii) affecting other tissues upon entering the systemic circulation. The latter includes crossing the blood–brain barrier, impacting the integrity thereof and, for instance, contributing to the biosynthesis of serotonin [2]. Additionally, higher fecal propionate levels were previously associated with longer uninterrupted infant sleep [34]. Another example of potential synergistic effects between GOS and nutrient blends was the strong stimulation of HICA in both age groups. Mechanistically, HICA is a metabolite of leucine, indicating that GOS shifts microbial fermentation from isovalerate (a BCFA) towards HICA. HICA is primarily produced by lactic acid bacteria like the bifidobacteria that were increased by GOS. Additionally, HICA was shown to have antimicrobial [35] and anti-inflammatory activity [36], while it can also improve muscle recovery [37]. Strong effects on N-acetylhistidine (infants) and pyridoxamine (young children) further highlight the potential of the combination of GOS with nutrient blends to maximize the metabolic activity of the gut microbiome. Pyridoxamine is a form of vitamin B6 that is critical in neurotransmitter synthesis (e.g., GABA, serotonin, dopamine) [38], suggesting that its microbial enhancement could influence early neural signaling pathways.

Further, for both age groups, BL (nutrient blend) increased indole-3-carboxaldehyde (tryptophan-derived metabolite linked to improved gut barrier function [39]), imidazole acetic acid (histidine-derived neuroregulatory metabolite [27]) and pipecolinic acid (lysine-derived linked to beneficial effects such as alleviating constipation [40,41]), along with levels of different B-vitamins (biotin, for infants only), pyridoxine and thiamine, highlighting the added value of providing nutrient blends in early life.

Despite age-related differences in microbiome composition, GOS consistently promoted *Bifidobacteriaceae* for both age groups and boosted the production of health-related SCFA, in line with earlier findings in children with a similar age range [16,42]. This demonstrates rather predictable outcomes, even if the starting microbiome composition is highly different. This bifidogenic effect is related to the stimulation of a broad range of (potential neuroactive) metabolites, including acetate, the key SCFA produced by *Bifidobacterium* species [43]. While *B. pseudocatenulatum* was the most abundant species in our dataset, other infant-associated *Bifidobacterium* species such as the infant-type bifidobacteria *B. longum*, *B. bifidum* and *B. breve* were also present and significantly promoted by GOS (with the exception of *B. breve* for young children). While general functions may be shared among all *Bifidobacterium* species (e.g., acetate and lactate production via the bifid shunt), others may be restricted to specific species. Other than SCFA, GOS, for instance, also stimulated tryptophan, tyrosine and phenylalanine-derived aromatic lactic acids, which are produced by infant-type *Bifidobacterium* species associated with breastfeeding, and have been linked to immune health and gut barrier function [13]. Especially, indole-3-propionic acid, a derivative of indole-3-lactic acid promoted by GOS, can readily enter the circulation and cross the blood–brain barrier and has been intensively studied for its neuroprotective effects [44]. In animal models, it promotes neurogenesis and recovery after axonal injury [45], while in humans, its levels are positively associated with brain-derived neurotrophic factor [46], which supports the growth, survival and differentiation of both developing and mature neurons. Additionally, indole-3-propionic acid produced by early-life gut microbiome is potentially critical in preventing allergic airway inflammation in adulthood [47]. Recent studies have demonstrated an association between the stimulation of *B. longum* and the increased production of indole-3-propionic acid [46,48], likely via promoting its precursor, indole-3-lactic acid. Interestingly, indole-3-lactic acid was stimulated by GOS for both age groups and has also been shown to exhibit neuroprotective effects [49]. Further, GOS also stimulated acetylagmatine (arginine metabolite), the acetylated form of agmatine that has neuroprotective effects against acute but also chronic neurodegenerative diseases [50]. In an animal model, agmatine was also implicated in mood regulation and synaptic plasticity, making it a compelling metabolite of interest in early-life interventions [51]. The acetylated form of spermidine (N-acetylspermidine) is also stimulated by GOS for young children. Spermidine, a polyamine derived from agmatine, has been shown to have protective effects against neuroinflammation and neural damage in different models [52,53,54]. Further, for young children, GOS also stimulated GABA production, a glutamate-derived, inhibitory neurotransmitter with relaxing, anti-anxiety and anti-convulsive effects [55]. Altogether, the stimulation of these metabolites highlights potential health benefits of GOS in pediatric nutrition.

Another notable finding was that butyrate production in infants and young children involved different taxa. For young children, butyrate production was positively associated with the presence of *Bifidobacterium pseudocatenulatum*, along with *Anaerobutyricum hallii*, a species that is unable to ferment oligosaccharides but can potently cross-feed with *Bifidobacterium* species to produce butyrate (via acetate or lactate) [56] or propionate (via 1,2-propanediol) [57]. In contrast to young children, *A. hallii* was absent in infant microbiota. Instead, individual infants showed butyrate production induced by GOS, potentially via the stimulation of *Clostridium neonatale,* as evidenced by its positive correlation with butyrate levels (Figure 5A). *C. neonatale* has not been demonstrated to cross-feed *Bifidobacterium* spp. to enhance butyrate synthesis. Altogether, these findings contribute to the literature on the succession of butyrate-producing taxa during infant gut microbiota development [58,59].

### 4.2. Strengths and Limitations of the Study

A notable advantage of the study’s approach is the utilization of the ex vivo SIFR^®^ technology, which has been validated to offer clinically predictive insights into gut microbiome modulation [19] and its subsequent effects on gut barrier integrity and immune function [20]. This technology facilitates a highly controlled research setting, allowing for any observed variations (except for minor technical variations) to be attributed to treatment effects. Furthermore, the SIFR^®^ technology enables the acquisition of insights into gut-derived metabolites, which are often challenging to analyze in clinical studies due to their rapid absorption or utilization in the body. This limitation confines many clinical studies to the analysis of fecal samples, which are excretion products from which most metabolites have been removed [60,61]. In this context, the present approach provides complementary mechanistic insight to clinical studies under more simplified, yet highly controlled ex vivo conditions. It should be noted that this ex vivo technology captured metabolite production at the level of the gut microbiota but did not account for subsequent processes such as absorption, systemic distribution or host-mediated metabolism. Importantly, the integration of metabolomics with microbiome profiling allowed us to identify a series of neuroactive or neuromodulatory metabolites (e.g., GABA, acetylagmatine, pyridoxamine, imidazoleacetic acid, pipecolinic acid, indole-3-propionic acid) that have been previously linked to gut–brain axis signaling, although the functional effects on neurodevelopment were thus not assessed in the present study. Nevertheless, this metabolite-level resolution is a notable strength, providing a mechanistic bridge between microbiota shifts and gut–brain axis modulation. Such mechanistic insights are particularly valuable for informing the design of future nutritional interventions targeting cognitive, emotional or behavioral outcomes in infants and young children. Nevertheless, despite the good congruence of data obtained with the SIFR^®^ technology and clinical findings [19,20], clinical studies are required to confirm the relevance of these observations for host health. The functional validation of neuroactive metabolites—e.g., their systemic bioavailability, CNS activity and impact on neurodevelopmental markers—remains a necessary step to move from correlation to causation.

While several of the identified metabolites, such as GABA, acetylagmatine, indole-3-propionic acid and pipecolinic acid, have been associated with neuroactive or neuroprotective functions in previous studies, these findings are primarily derived from animal models or adult populations. Their relevance in the context of early-life development remains uncertain as factors such as the maturation state of the blood–brain barrier, systemic bioavailability of microbiome-derived metabolites, as well as age-specific neurophysiology, may influence their potential effects in infants and young children.

A critical side note on the impact of GOS is that while GOS generally exerted potent bifidogenic effects in each age group, one specific infant and young child did not display bifidogenic effects. Instead, an increase of *Enterobacteriaceae* was noted for these subjects. This suggests that strategies aimed at promoting bifidobacterial colonization, such as the inclusion of *Bifidobacterium* probiotics or symbiotic approaches, could be explored to also consolidate bifidogenic effects for such donors with depleted *Bifidobacterium* levels at baseline. However, this remains speculative and was not directly assessed in the present study. This observation also highlights a limitation of generalizing prebiotic effects across all infants and suggests that personalized or combined prebiotic–probiotic strategies may be more effective in achieving desired neurodevelopmental outcomes.

The final limitation of the study is the relatively small number of donors per age group (*n* = 6). While this sample size may appear limited, it reflects the minimal recommended design for the SIFR^®^ ex vivo fermentation platform, for which this number of subjects has previously been shown to generate microbiome modulation insights that are predictive of findings from clinical studies, down to the species level [19]. Earlier in vitro gut microbiome studies frequently relied on a single donor microbiota [62], whereas the SIFR^®^ technology was specifically developed to enable parallel testing of multiple independent donors. However, the limited sample size may restrict the detection of subtle effects, and the use of a more lenient significance threshold (adjusted *p* < 0.20) increases the likelihood of false-positive findings. Therefore, the reported associations should be interpreted as exploratory and hypothesis-generating rather than confirmatory and require validation in larger cohorts or clinical studies. In addition, given the substantial interindividual variability, particularly among infants, larger numbers of donors would improve interpretability and generalizability.

### 4.3. Potential Synergistic Effects Between GOS and Nutrient Blends

From a formulation perspective, the present findings suggest that GOS is a robust baseline intervention due to its consistent bifidogenic effects and associated metabolite production across age groups. The addition of nutrient blends further modulated microbial metabolism and enabled additional effects on selected metabolites, including potential synergistic responses. This indicates that combining fermentable substrates with targeted micronutrients and amino acids is a promising strategy to optimize microbiome-targeted interventions. However, the optimal composition of such combinations is likely dependent on microbiome maturity and the desired functional outputs. These results therefore support the use of ex vivo platforms such as the SIFR^®^ technology platform as research tools to guide the design and optimization of next-generation nutritional formulations, which should subsequently be validated in clinical studies.

Of particular interest was that the combination of GOS with nutrient blends resulted in pronounced effects on specific metabolites, most notably propionate in infants and 2-hydroxyisocaproic acid (HICA) in both age groups. These findings suggest that combining fermentable substrates with micronutrients and amino acids can enhance microbial metabolic activity beyond additive effects. Mechanistically, such synergies may arise from shifts in microbial community composition, particularly the stimulation of bifidobacteria, as well as from increased availability of precursor compounds for microbial metabolism. Future studies could explore whether targeted inclusion of additional precursor substrates further enhances the production of selected microbiome-derived metabolites.

## 5. Conclusions

GOS alone and in combination with nutrient blends modulated microbiome composition and metabolite production in an age-dependent manner under ex vivo conditions. Several microbiome-derived metabolites associated with gut–brain axis signaling were affected, including GABA, acetylagmatine and HICA. While these findings provide mechanistic insights into how nutritional interventions may influence microbiome-derived metabolites during early life, their potential impact on neurodevelopment remains to be established. Future studies, particularly in clinical settings, are required to determine the relevance of these observations in vivo.

## Figures and Tables

**Table 1:** Nutritional composition (g/L) of the blends of vitamins, micronutrients and amino acids for each age group.

Ingredient	BL (Infants)	BL+ (Young Children)
Vitamin B1	0.00115	0.00027
Vitamin B2	0.00175	0.00040
Vitamin B6	0.00158	0.00036
Zinc	0.00936	0.00215
Iron	0.01458	0.00334
Copper	0.00095	0.00022
Histidine	0.36728	0.08429
Isoleucine	0.88298	0.20265
Lysine	0.88261	0.20257
Leucine	1.59484	0.36603
Total	3.75708	0.86228

## Data Availability

The datasets generated during and/or analyzed during the current study are available from the corresponding author upon reasonable request.

## Supplementary Materials

The following supporting information can be downloaded at: https://www.mdpi.com/article/10.3390/metabo16040255/s1, Table S1: Cell density measured by flow cytometry and the absolute abundances (cells/mL) of bacterial families and OTUs in the samples of infants’ and young children’s microbiota; Table S2: Statistical analysis results for bacterial OTUs in the infants’ and young children’s microbiota; Table S3: Canonical correlations calculated via Regularized Canonical Correlation Analysis for the three main SCFAs and the bacterial OTUs in Figure 5; Table S4: Statistical analysis results for metabolites identified via untargeted metabolite profiling (LC-MS/MS).

## Abbreviations

The following abbreviations are used in this manuscript:

SCFA	Short-chain fatty acid
BCFA	Branched-chain fatty acid
SIFR^®^	Systemic Intestinal Fermentation Research
GOS	Galacto-oligosaccharides
HICA	2-Hydroxyisocaproic acid
GABA	γ-Aminobutyric acid
MAMP	Microorganism-associated molecular patterns
BL	Nutrient blend
GB	GOS combined with nutrient blend
NSC	No-substrate control
OTU	Operational taxonomic unit

## References

[1] Carabotti M., Scirocco A., Maselli M.A., Severi C. (2015). The Gut-Brain Axis: Interactions between Enteric Microbiota, Central and Enteric Nervous Systems. Ann. Gastroenterol. Q. Publ. Hell. Soc. Gastroenterol..

[2] Silva Y.P., Bernardi A., Frozza R.L. (2020). The Role of Short-Chain Fatty Acids From Gut Microbiota in Gut-Brain Communication. Front. Endocrinol..

[3] Gao K., Mu C., Farzi A., Zhu W. (2020). Tryptophan Metabolism: A Link Between the Gut Microbiota and Brain. Adv. Nutr..

[4] Morais L.H., Schreiber H.L., Mazmanian S.K. (2021). The Gut Microbiota–Brain Axis in Behaviour and Brain Disorders. Nat. Rev. Microbiol..

[5] Martin C.R., Osadchiy V., Kalani A., Mayer E.A. (2018). The Brain-Gut-Microbiome Axis. Cell. Mol. Gastroenterol. Hepatol..

[6] Cerdó T., Diéguez E., Campoy C. (2020). Impact of Gut Microbiota on Neurogenesis and Neurological Diseases during Infancy. Curr. Opin. Pharmacol..

[7] Laursen M.F., Bahl M.I., Michaelsen K.F., Licht T.R. (2017). First Foods and Gut Microbes. Front. Microbiol..

[8] Derrien M., Alvarez A.-S., de Vos W.M. (2019). The Gut Microbiota in the First Decade of Life. Trends Microbiol..

[9] Fattorusso A., Genova L.D., Dell’Isola G.B., Mencaroni E., Esposito S. (2019). Autism Spectrum Disorders and the Gut Microbiota. Nutrients.

[10] Hill C., Guarner F., Reid G., Gibson G.R., Merenstein D.J., Pot B., Morelli L., Canani R.B., Flint H.J., Salminen S. (2014). The International Scientific Association for Probiotics and Prebiotics Consensus Statement on the Scope and Appropriate Use of the Term Probiotic. Nat. Rev. Gastroenterol. Hepatol..

[11] Pärtty A., Kalliomäki M., Wacklin P., Salminen S., Isolauri E. (2015). A Possible Link between Early Probiotic Intervention and the Risk of Neuropsychiatric Disorders Later in Childhood: A Randomized Trial. Pediatr. Res..

[12] Gibson G.R., Hutkins R., Sanders M.E., Prescott S.L., Reimer R.A., Salminen S.J., Scott K., Stanton C., Swanson K.S., Cani P.D. (2017). Expert Consensus Document: The International Scientific Association for Probiotics and Prebiotics (ISAPP) Consensus Statement on the Definition and Scope of Prebiotics. Nat. Rev. Gastroenterol. Hepatol..

[13] Laursen M.F., Sakanaka M., von Burg N., Mörbe U., Andersen D., Moll J.M., Pekmez C.T., Rivollier A., Michaelsen K.F., Mølgaard C. (2021). Bifidobacterium Species Associated with Breastfeeding Produce Aromatic Lactic Acids in the Infant Gut. Nat. Microbiol..

[14] Turroni F., Milani C., Ventura M., van Sinderen D. (2022). The Human Gut Microbiota during the Initial Stages of Life: Insights from Bifidobacteria. Curr. Opin. Biotechnol..

[15] Vandenplas Y., Greef E.D., Veereman G. (2014). Prebiotics in Infant Formula. Gut Microbes.

[16] Ambrogi V., Bottacini F., Cao L., Kuipers B., Schoterman M., van Sinderen D. (2023). Galacto-Oligosaccharides as Infant Prebiotics: Production, Application, Bioactive Activities and Future Perspectives. Crit. Rev. Food Sci. Nutr..

[17] Bakshi S., Paswan V.K., Yadav S.P., Bhinchhar B.K., Kharkwal S., Rose H., Kanetkar P., Kumar V., Al-Zamani Z.A.S., Bunkar D.S. (2023). A Comprehensive Review on Infant Formula: Nutritional and Functional Constituents, Recent Trends in Processing and Its Impact on Infants’ Gut Microbiota. Front. Nutr..

[18] Kanellopoulos A.K., Costello S., Mainardi F., Koshibu K., Deoni S., Schneider N. (2023). Dynamic Interplay between Social Brain Development and Nutrient Intake in Young Children. Nutrients.

[19] Van den Abbeele P., Deyaert S., Thabuis C., Perreau C., Bajic D., Wintergerst E., Joossens M., Firrman J., Walsh D., Baudot A. (2023). Bridging Preclinical and Clinical Gut Microbiota Research Using the Ex Vivo SIFR^®^ Technology. Front. Microbiol..

[20] Van den Abbeele P., Kunkler C.N., Poppe J., Rose A., van Hengel I.A.J., Baudot A., Warner C.D. (2024). Serum-Derived Bovine Immunoglobulin Promotes Barrier Integrity and Lowers Inflammation for 24 Human Adults Ex Vivo. Nutrients.

[21] Van den Abbeele P., Deyaert S., Albers R., Baudot A., Mercenier A. (2023). Carrot RG-I Reduces Interindividual Differences between 24 Adults through Consistent Effects on Gut Microbiota Composition and Function Ex Vivo. Nutrients.

[22] Van den Abbeele P., Poppe J., Deyaert S., Laurie I., Otto Gravert T.K., Abrahamsson A., Baudot A., Karnik K., Risso D. (2023). Low-No-Calorie Sweeteners Exert Marked Compound-Specific Impact on the Human Gut Microbiota Ex Vivo. Int. J. Food Sci. Nutr..

[23] Benjamini Y., Hochberg Y. (1995). Controlling the False Discovery Rate: A Practical and Powerful Approach to Multiple Testing. J. R. Stat. Soc. Ser. B.

[24] Rohart F., Gautier B., Singh A., Cao K.-A.L. (2017). mixOmics: An R Package for ‘omics Feature Selection and Multiple Data Integration. PLoS Comput. Biol..

[25] Chen C., Xiao Q., Wen Z., Gong F., Zhan H., Liu J., Li H., Jiao Y. (2025). Gut Microbiome-Derived Indole-3-Carboxaldehyde Regulates Stress Vulnerability in Chronic Restraint Stress by Activating Aryl Hydrocarbon Receptors. Pharmacol. Res..

[26] Tunnicliff G. (1998). Pharmacology and Function of Imidazole 4-Acetic Acid in Brain. Gen. Pharmacol. Vasc. Syst..

[27] Prell G.D., Martinelli G.P., Holstein G.R., Matulić-Adamić J., Watanabe K.A., Chan S.L.F., Morgan N.G., Haxhiu M.A., Ernsberger P. (2004). Imidazoleacetic Acid-Ribotide: An Endogenous Ligand That Stimulates Imidazol(in)e Receptors. Proc. Natl. Acad. Sci. USA.

[28] Matsumoto S., Yamamoto S., Sai K., Maruo K., Adachi M., Saitoh M., Nishizaki T. (2003). Pipecolic Acid Induces Apoptosis in Neuronal Cells. Brain Res..

[29] Gutierrez M.D.C., Giacobini E. (1985). Identification and Characterization of Pipecolic Acid Binding Sites in Mouse Brain. Neurochem. Res..

[30] Tannock G.W., Lee P.S., Wong K.H., Lawley B. (2016). Why Don’t All Infants Have Bifidobacteria in Their Stool?. Front. Microbiol..

[31] Van den Abbeele P., Ghyselinck J., Marzorati M., Koch A.-M., Lambert W., Michiels J., Chalvon-Demersay T. (2022). The Effect of Amino Acids on Production of SCFA and bCFA by Members of the Porcine Colonic Microbiota. Microorganisms.

[32] Barker H.A. (1981). Amino Acid Degradation by Anaerobic Bacteria. Annu. Rev. Biochem..

[33] Ueki A., Goto K., Ohtaki Y., Kaku N., Ueki K. (2017). Description of *Anaerotignum aminivorans* Gen. Nov., Sp. Nov., a Strictly Anaerobic, Amino-Acid-Decomposing Bacterium Isolated from a Methanogenic Reactor, and Reclassification of *Clostridium 
propionicum*, *Clostridium neopropionicum* and *Clostridium lactatifermentans* as Species of the Genus *Anaerotignum*. Int. J. Syst. Evol. Microbiol..

[34] Heath A.-L.M., Haszard J.J., Galland B.C., Lawley B., Rehrer N.J., Drummond L.N., Sims I.M., Taylor R.W., Otal A., Taylor B. (2020). Association between the Faecal Short-Chain Fatty Acid Propionate and Infant Sleep. Eur. J. Clin. Nutr..

[35] Sakko M., Tjäderhane L., Sorsa T., Hietala P., Järvinen A., Bowyer P., Rautemaa R. (2012). 2-Hydroxyisocaproic Acid (HICA): A New Potential Topical Antibacterial Agent. Int. J. Antimicrob. Agents.

[36] Nieminen M.T., Hernandez M., Novak-Frazer L., Kuula H., Ramage G., Bowyer P., Warn P., Sorsa T., Rautemaa R. (2014). Dl-2-Hydroxyisocaproic Acid Attenuates Inflammatory Responses in a Murine Candida Albicans Biofilm Model. Clin. Vaccine Immunol..

[37] Lang C.H., Pruznak A., Navaratnarajah M., Rankine K.A., Deiter G., Magne H., Offord E.A., Breuillé D. (2013). Chronic α-Hydroxyisocaproic Acid Treatment Improves Muscle Recovery after Immobilization-Induced Atrophy. Am. J. Physiol.-Endocrinol. Metab..

[38] Parra M., Stahl S., Hellmann H. (2018). Vitamin B6 and Its Role in Cell Metabolism and Physiology. Cells.

[39] Li X., Zhang B., Hu Y., Zhao Y. (2021). New Insights into Gut-Bacteria-Derived Indole and Its Derivatives in Intestinal and Liver Diseases. Front. Pharmacol..

[40] Li H., Xiao H., Yuan L., Yan B., Pan Y., Tian P., Zhang W. (2023). Protective Effect of L-Pipecolic Acid on Constipation in C57BL/6 Mice Based on Gut Microbiome and Serum Metabolomic. BMC Microbiol..

[41] Ou Y., Chen S., Ren F., Zhang M., Ge S., Guo H., Zhang H., Zhao L. (2019). Lactobacillus Casei Strain Shirota Alleviates Constipation in Adults by Increasing the Pipecolinic Acid Level in the Gut. Front. Microbiol..

[42] Giovannini M., Verduci E., Gregori D., Ballali S., Soldi S., Ghisleni D., Riva E., PLAGOS Trial Study Group (2014). Prebiotic Effect of an Infant Formula Supplemented with Galacto-Oligosaccharides: Randomized Multicenter Trial. J. Am. Coll. Nutr..

[43] De Vuyst L., Moens F., Selak M., Rivière A., Leroy F. (2014). Summer Meeting 2013: Growth and Physiology of Bifidobacteria. J. Appl. Microbiol..

[44] Jiang H., Chen C., Gao J. (2022). Extensive Summary of the Important Roles of Indole Propionic Acid, a Gut Microbial Metabolite in Host Health and Disease. Nutrients.

[45] Serger E., Luengo-Gutierrez L., Chadwick J.S., Kong G., Zhou L., Crawford G., Danzi M.C., Myridakis A., Brandis A., Bello A.T. (2022). The Gut Metabolite Indole-3 Propionate Promotes Nerve Regeneration and Repair. Nature.

[46] Kim C.-S., Jung S., Hwang G.-S., Shin D.-M. (2023). Gut Microbiota Indole-3-Propionic Acid Mediates Neuroprotective Effect of Probiotic Consumption in Healthy Elderly: A Randomized, Double-Blind, Placebo-Controlled, Multicenter Trial and in Vitro Study. Clin. Nutr..

[47] Perdijk O., Butler A., Macowan M., Chatzis R., Bulanda E., Grant R.D., Harris N.L., Wypych T.P., Marsland B.J. (2024). Antibiotic-Driven Dysbiosis in Early Life Disrupts Indole-3-Propionic Acid Production and Exacerbates Allergic Airway Inflammation in Adulthood. Immunity.

[48] Mercenier A., Vu L.D., Poppe J., Albers R., McKay S., Van den Abbeele P. (2024). Carrot-Derived Rhamnogalacturonan-I Consistently Increases the Microbial Production of Health-Promoting Indole-3-Propionic Acid Ex Vivo. Metabolites.

[49] Wong C.B., Tanaka A., Kuhara T., Xiao J. (2020). Potential Effects of Indole-3-Lactic Acid, a Metabolite of Human Bifidobacteria, on NGF-Induced Neurite Outgrowth in PC12 Cells. Microorganisms.

[50] Xu W., Gao L., Li T., Shao A., Zhang J. (2018). Neuroprotective Role of Agmatine in Neurological Diseases. Curr. Neuropharmacol..

[51] Meylan E.M., Breuillaud L., Seredenina T., Magistretti P.J., Halfon O., Luthi-Carter R., Cardinaux J.-R. (2016). Involvement of the Agmatinergic System in the Depressive-like Phenotype of the Crtc1 Knockout Mouse Model of Depression. Transl. Psychiatry.

[52] Madeo F., Eisenberg T., Pietrocola F., Kroemer G. (2018). Spermidine in Health and Disease. Science.

[53] Yang Y., Chen S., Zhang Y., Lin X., Song Y., Xue Z., Qian H., Wang S., Wan G., Zheng X. (2017). Induction of Autophagy by Spermidine Is Neuroprotective via Inhibition of Caspase 3-Mediated Beclin 1 Cleavage. Cell Death Dis..

[54] Qi L., Zhang X., Liu Y., Guo P., Siddique R., Mazhar M., Xue S., Yong V.W., Xue M. (2025). Spermidine Exerts Neuroprotective Effects Following Intracerebral Hemorrhage in Mice Through Anti-Inflammation and Blood-Brain Barrier Protection. J. Inflamm. Res..

[55] Foster A.C., Kemp J.A. (2006). Glutamate- and GABA-Based CNS Therapeutics. Curr. Opin. Pharmacol..

[56] Shetty S.A., Zuffa S., Bui T.P.N., Aalvink S., Smidt H., De Vos W.M. (2018). Reclassification of *Eubacterium hallii* as *Anaerobutyricum hallii* Gen. Nov., Comb. Nov., and Description of *Anaerobutyricum soehngenii* Sp. Nov., a Butyrate and Propionate-Producing Bacterium from Infant Faeces. Int. J. Syst. Evol. Microbiol..

[57] Engels C., Ruscheweyh H.-J., Beerenwinkel N., Lacroix C., Schwab C. (2016). The Common Gut Microbe Eubacterium Hallii Also Contributes to Intestinal Propionate Formation. Front. Microbiol..

[58] Appert O., Garcia A.R., Frei R., Roduit C., Constancias F., Neuzil-Bunesova V., Ferstl R., Zhang J., Akdis C., Lauener R. (2020). Initial Butyrate Producers during Infant Gut Microbiota Development Are Endospore Formers. Environ. Microbiol..

[59] Nilsen M., Madelen Saunders C., Leena Angell I., Arntzen M.Ø., Lødrup Carlsen K.C., Carlsen K.-H., Haugen G., Hagen L.H., Carlsen M.H., Hedlin G. (2020). Butyrate Levels in the Transition from an Infant- to an Adult-Like Gut Microbiota Correlate with Bacterial Networks Associated with Eubacterium Rectale and Ruminococcus Gnavus. Genes.

[60] Ruppin H., Bar-Meir S., Soergel K.H., Wood C.M., Schmitt M.G. (1980). Absorption of Short-Chain Fatty Acids by the Colon. Gastroenterology.

[61] Delcour J.A., Aman P., Courtin C.M., Hamaker B.R., Verbeke K. (2016). Prebiotics, Fermentable Dietary Fiber, and Health Claims. Adv. Nutr..

[62] Van den Abbeele P., Sprenger N., Ghyselinck J., Marsaux B., Marzorati M., Rochat F. (2021). A Comparison of the In Vitro Effects of 2’Fucosyllactose and Lactose on the Composition and Activity of Gut Microbiota from Infants and Toddlers. Nutrients.

